# Acute annular outer retinopathy preceded by invasive ductal breast carcinoma: a case report

**DOI:** 10.1186/s12886-022-02647-w

**Published:** 2022-11-24

**Authors:** Rishi B. Gupta, Harry Dang, Danah Albreiki, Michael LE. Dollin, Bonnie Weston, Chloe C. Gottlieb

**Affiliations:** 1 Faculty of Medicine, University of Ottawa, 451 Smyth Road, Ottawa, ON Canada; 2 Department of Ophthalmology & Visual Sciences, Dalhousie University, Halifax, NS Canada; 3 The University of Ottawa Eye Institute, Ottawa, ON Canada; 4 The Ottawa Hospital Research Institute, Ottawa, ON Canada

**Keywords:** Acute annular outer retinopathy, Acute zonal occult outer retinopathy, Invasive ductal carcinoma, Retinal disease, Case report

## Abstract

**Background:**

Acute annular outer retinopathy (AAOR) is an uncommon disease. To date, there are few documented cases in the literature. Our case report is the first to describe a case of acute annular outer retinopathy in a patient with invasive ductal breast carcinoma.

**Case presentation:**

The patient presented with photopsias and visual loss approximately 3 weeks prior to a diagnosis of invasive ductal breast carcinoma. We have documented the outer annular white ring seen in the acute phase of this disease and correlate it anatomically with Spectral-domain optical coherence tomography (SD-OCT) imaging. We identified RPE atrophy with nodular hyperreflectivity and loss of ellipsoid layer within the white annular ring with corresponding visual field loss. Fundus autofluorescence correlated with structural alterations seen on SD-OCT and showed both presumed active hyperautofluorescent zones with patchy hypoautofluorescent zones of atrophy and a classic annular hyperautofluorescent border. This case provides additional information about the natural history of this rare entity and its prognosis and varied presentation.

**Conclusions:**

The authors report a single case of acute annular outer retinopathy in a patient with invasive ductal breast carcinoma with the corresponding SD-OCT, fundus autofluorescence and visual field findings, during the acute phase of the disease. These findings provide new insight into the characteristic features, etiology and progression of this rare disease.

## Background

Acute annular outer retinopathy (AAOR) is a rare condition of unknown etiology and is considered to be a variant of acute zonal occult outer retinopathy (AZOOR) [1]. There are only 13 cases that have been reported in the literature to date. This disease is characterized by an irregular annular band of deep retinal opacification in a peripapillary location. It is grey-white in color and is associated with a symptomatic visual field defect. This case of acute annular outer retinopathy presented in a patient with invasive ductal breast carcinoma.

## Case presentation

A 66-year-old Caucasian woman presented with a two-week history of bilateral sequential subacute progressive visual field loss and photopsias initially affecting the left eye and rapidly progressing to the right eye within several days. The patient denied prodromal symptoms and there were no recent infections, no recent travel history or sick contacts. The past medical history was significant for hypothyroidism, migraines, hearing loss and psoriasis. The past surgical history included a bilateral mammoplasty, tubal ligation, and a cone biopsy of the cervix done 40 years ago that revealed pre-cancerous changes. A recent Pap smear was suspicious for malignancy. Attempted repeat biopsy 1 week prior to presentation failed due to scarring of the cervix. Medications included levothyroxine, zopiclone, and an estradiol vaginal ring. The patient had no known drug allergies. The family history was positive for breast cancer. There was no significant past ocular history except for myopia.

On initial ophthalmic examination by the referring physician, best-corrected visual acuity (BCVA) was 20/30 in the right eye and 20/70 in the left eye. Pupils were equal and reactive with no afferent pupillary defect. Confrontation visual fields revealed bilateral enlargement of the blind spots. Color vision was 7/9 in the right eye and 0/9 in the left eye. There were rare anterior chamber cells in both eyes. Intraocular pressures were 17 mmHg bilaterally. Fundus exam revealed normal optic nerve heads with no swelling or vascular engorgement. There was no significant vitritis. Surrounding each disc was a contiguous area of retinal atrophy and retinal pigment epithelium (RPE) disruption bordered by a continuous leading edge of elevated white retina. This bisected the fovea in the left eye and was bordering the fovea in the right eye. An infectious disease workup was initiated. However, due to the proximity of the leading edge to the fovea in the right eye, oral prednisone 100 mg daily was started immediately.

The patient was examined two days later. Despite the large doses of prednisone, BCVA had dropped to 20/400 in the right eye and 20/400 in the left eye. There was again no significant intraocular inflammation. The white line of elevated retina and corresponding area of retinal atrophy had enlarged in both eyes, and had now crossed the fovea in the right eye. Due to the unusual location and pattern, an autoimmune process was suspected. Considering the possible history of gynecological malignancy, arrangements were made for hospital admission and workup.

**Fig. 1:**
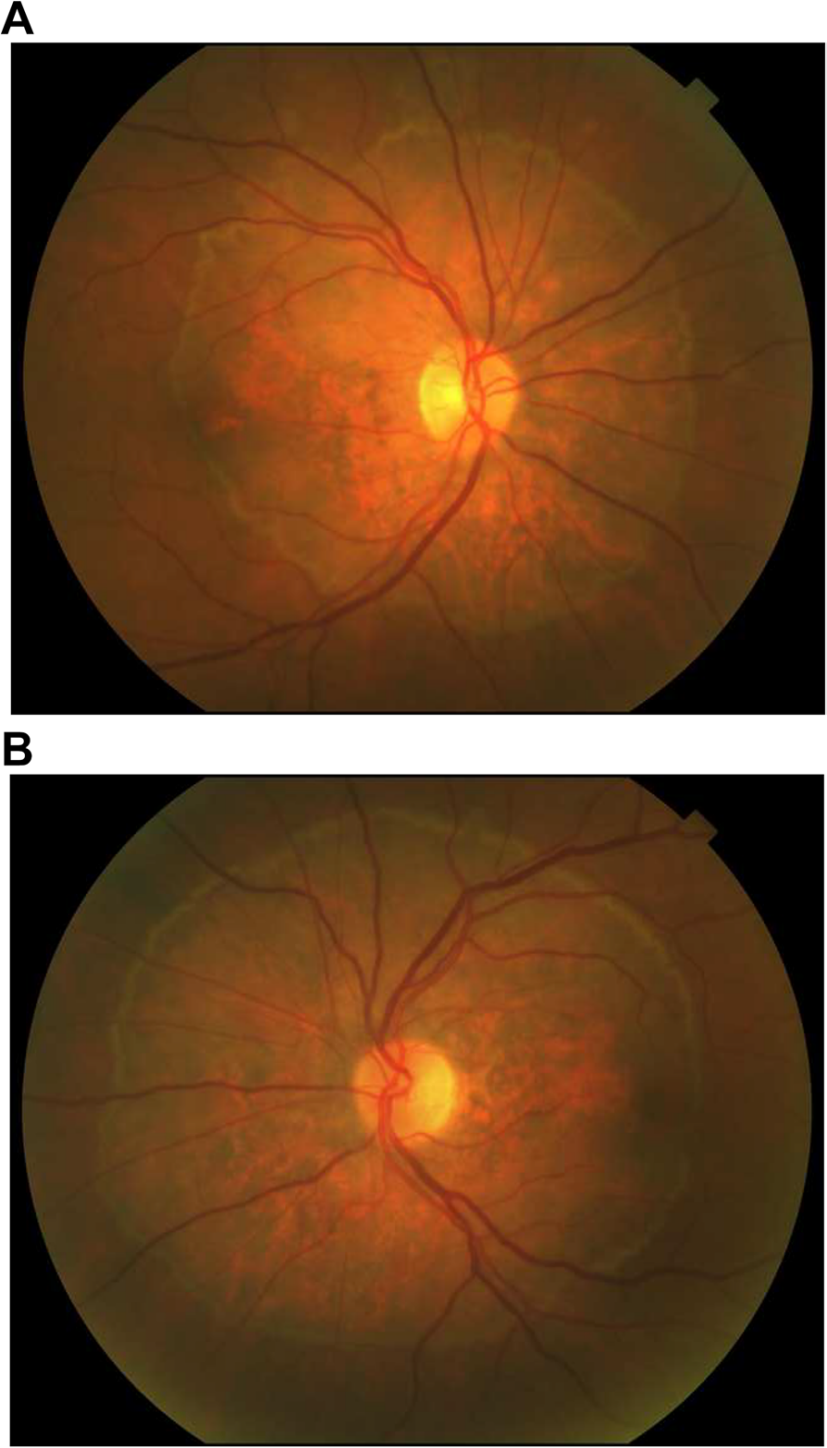
Fundus examination of the right eye (**A**) and the left eye (**B**) showing a classic pattern of symmetrical bilateral retinal atrophy surrounding each optic nerve. The outer limit of the retinal atrophy showed a distinctive and slightly irregular white border

On initial examination at our institution, the BCVA was 20/500 in the right eye and count-fingers at 1 foot in the left eye. The intraocular pressures were normal. Pupils were 5 mm bilaterally and reactive to light, both direct and consensual. There was no afferent pupillary defect. Color vision was reduced with Ishihara plate testing 1/16 in the right eye and 2/16 in the left eye. Confrontation visual fields were unremarkable. Extraocular movements were normal. Ductions, versions and smooth pursuit were normal. No nystagmus was noted. The anterior segment was normal in both eyes. A grade 2 + nuclear sclerotic cataract was noted in both eyes. The vitreous had no cells and no haze. Fundus examination of the left and right eye showed a classic pattern of symmetrical bilateral retinal atrophy surrounding each optic nerve (Fig. 1). The outer limit of the retinal atrophy showed a distinctive and slightly irregular white border. The area within the atrophic retina showed scattered zones of RPE clumping/migration. The peripheral retinal examination showed no retinal or choroidal lesions, and no vascular changes. The optic nerves appeared normal with no swelling, atrophy or cupping.

**Fig. 2:**
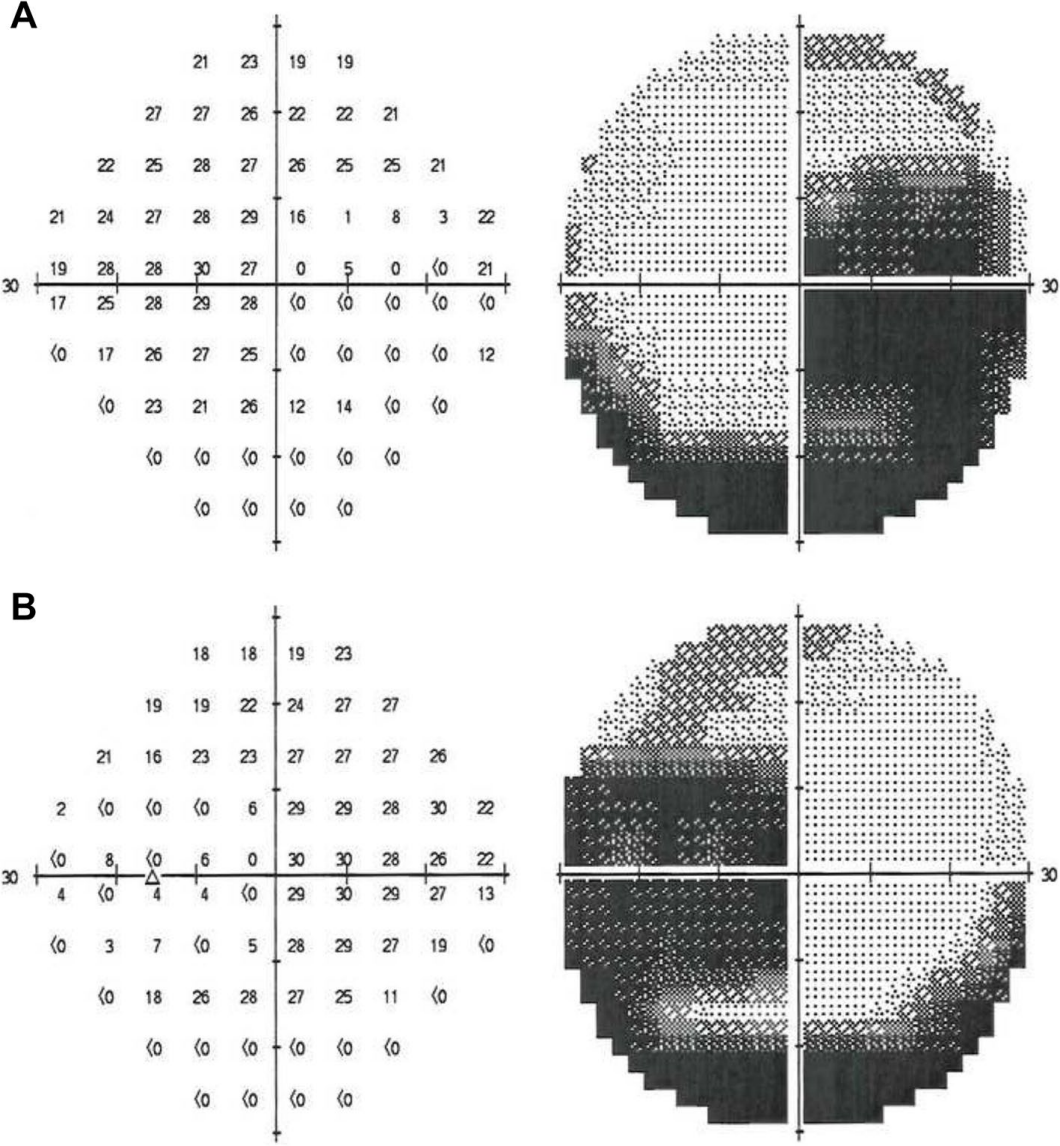
Humphrey visual field testing central 30–2 white stimulus SITA-standard protocol demonstrating symmetrical enlargement of the blind spots with inferior visual field loss in the right eye (**A**) and the left eye (R)

**Fig. 3:**
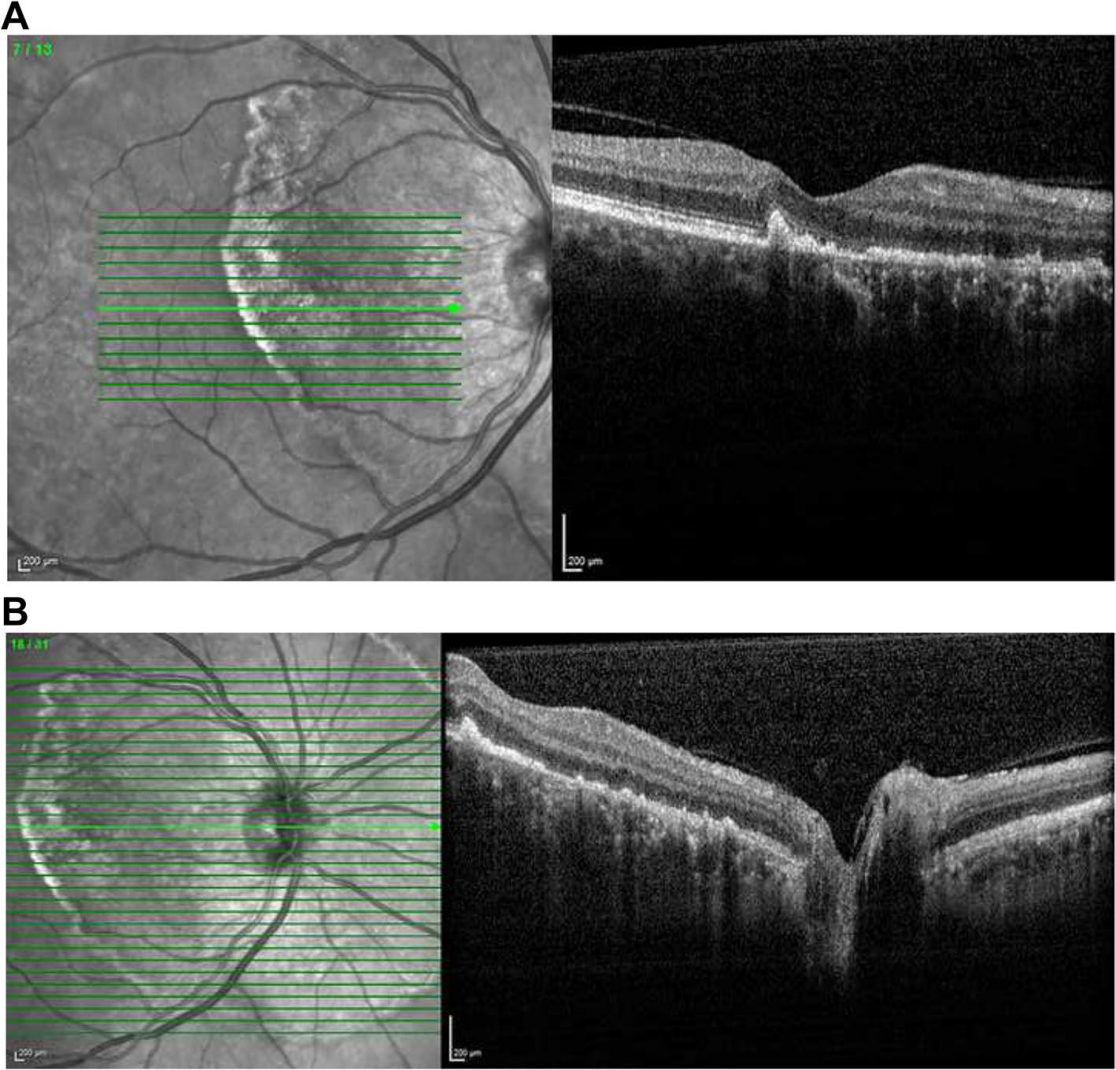
Spectral-domain OCT of the macular (**A**) and the optic nerve head (**B**) of the right eye showing a normal retinal architecture outside the zone of peripapillary atrophy. An abrupt and elevated nodular disruption of the RPE at the margin of the annulus can be seen, located just beyond the temporal edge of the fovea. Patchy RPE loss and destruction of the ellipsoid layer are seen within the peripapillary atrophied zone extending to the margin of the optic disc

**Fig. 4:**
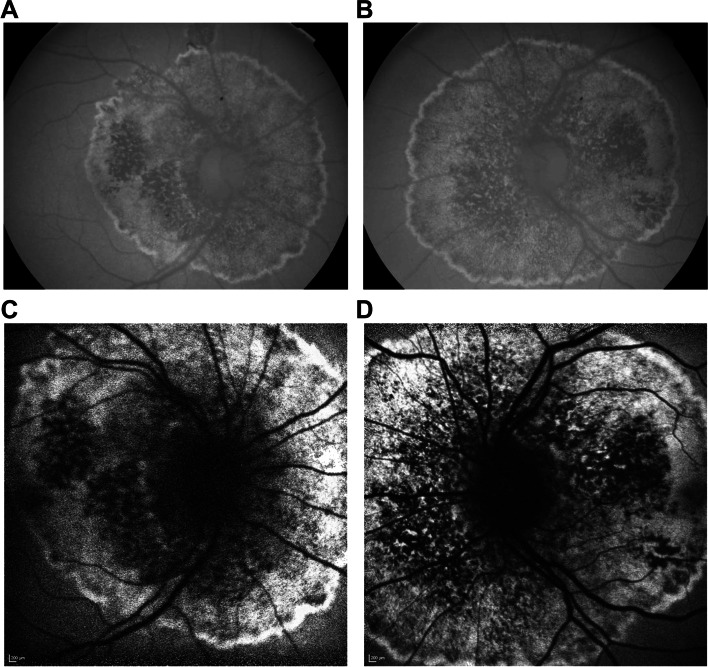
Fundus autofluorescence images of the right and left eye using Topcon 3D OCT-2000 (**A** and **B**, respectively) and using Heidelberg Spectralis OCT (**C** and **D**, respectively) showing both presumed active hyperautofluorescent zones with patchy hypoautofluorescent zones of atrophy and a classic annular hyperautofluorescent border

Humphrey visual field testing central 30–2 white stimulus SITA-standard protocol demonstrated symmetrical enlargement of the blind spots with inferior visual field loss corroborating the retinal changes (Fig. 2). Spectral-domain optical coherence tomography (SD-OCT) showed a normal retinal architecture outside the zone of peripapillary atrophy. There was an abrupt and elevated nodular disruption of the RPE at the margin of the annulus, located just beyond the temporal edge of the fovea. Patchy RPE loss and destruction of the ellipsoid layer were seen within the peripapillary atrophied zone extending to the margin of the optic disc (Fig. 3). The structural abnormalities seen on SD-OCT correlated with findings on fundus autofluorescence which showed a classic annular hyperautofluorescent border with presumed active hyperautofluorescent zones with patchy hypoautofluorescent zones of atrophy (Fig. 4).

Laboratory analyses including erythrocyte sedimentation rate, C-reactive protein, complete blood count with differential, antinuclear antibody, anti-neutrophil cytoplasmic antibodies, rheumatoid factor and angiotensin-converting enzyme revealed normal findings. Human Immunodeficiency Virus (HIV), Herpes Simplex Virus (HSV), Lyme disease (*Borrelia burgdorferi*), and *Bartonella* (Cat Scratch Disease) serologies were negative. Varicella Zoster Virus (VZV) immunoglobulin G (IgG) was positive. Toxoplasmosis immunoglobulin G (IgG) and immunoglobulin M (IgM) were nonreactive. An MRI with contrast of the orbits showed no optic nerve pathology.

The patient was started on 1 g IV methylprednisolone for three days followed by oral prednisone 100 mg daily. Two days after presentation, visual acuity was CF at 1 foot in both the right and left eye. There were no changes to the white annular band. Two weeks after presentation, visual acuity declined to HM in the right eye and CF at 1 foot in the left eye. Retinal pigment epithelial atrophy persisted in both eyes and there was no detectable progression of the annular band in both eyes. Three months after presentation, the best-corrected visual acuity was 20/200 in the right eye and CF at 1 foot in the left eye. The annular band remained unchanged in both eyes.

An extensive workup was performed to assess for an occult neoplasm, including Magnetic Resonance Imaging (MRI) scans of the brain, pelvis, spine and breast, and Computed Tomography (CT) scans of the chest, abdomen, pelvis and head. The investigations detected an invasive ductal carcinoma of the left breast that was subsequently confirmed to be of Histological Grade/Nottingham Score of 2/3 following an ultrasound-guided core needle biopsy of the breast.

## Discussion and conclusions

The etiology of acute annular outer retinopathy is still uncertain. Luckie et al. reported visual improvement in a case of uniocular acute progressive outer retinitis following treatment with intravenous acyclovir [2]. This case was thought to be secondary to a herpes virus infection. The authors described that the slow response to intravenous acyclovir in this case suggested that the infection was likely from the Epstein-Barr virus or varicella zoster virus, because ID50 level was high for both viruses [2]. Our patient was negative for IgM antibodies against herpes simplex virus, Toxoplasmosis, Bartonella, syphilis, Lyme and varicella zoster virus (VZV IgG positive). CMV serology was not performed. Fungal infection has been implicated with previous case reports and reportedly treatment with antifungal therapy has been effective (PMID: 15,695,657).

Gass initially postulated that the cause of acute damage in AAOR is due to an immune reaction triggering a humoral or cellular response to an occult infection of viral etiology [1]. The authors suggested that the formation of a white, annular outer retinal ring is a result of an immune response to a virus and that the papillocentric configuration of the white annular ring may allow the virus to gain access to the neurosensory retina from the central nervous system via the optic nerve [1, 3]. Unlike the case described by Mitamura et al. [4] and one of the four cases reported by Fekrat et al. [3], our patient developed sequelae and had a poor visual recovery. Gass and Stern hypothesized that there would be no ophthalmoscopic sequelae if receptor cells recover their function [1]. However, there may be retinal pigment epithelial atrophy and progressive outer retinal degeneration if receptor cells permanently lose their function. In the case reported by Mitamura et al., the authors suspected a recovery of receptor cell function after multifocal electroretinogram indicated a significant improvement over a 1-year period [4].

In our case, there was no evidence of infection and the postulated association with the patient’s recent diagnosis of invasive ductal breast carcinoma suggests an autoimmune etiology. Given the close temporal association of the ductal carcinoma with the onset of visual symptoms, this case could present an association between cancer and AAOR.

Fekrat et al. described two cases of AAOR that responded to systemic corticosteroids [3]. A separate group subsequently reported a diminished annular lesion following treatment with acyclovir and oral prednisone over a period of 6 weeks [5]. In their patient, systemic administration of steroids was considered but not recommended because the patient had an increased risk of developing an infection secondary to pneumoconiosis [5]. In our patient, inflammatory cells were undetected in both the vitreous and the anterior chamber, and an association with an infectious etiology was considered and thought to less likely than the association with neoplastic disease.

Our patient was similar to Patients 2 and 4 described by Fekrat et al. [3], and to the cases described by Harino et al. [6] and Rodriguez-Coleman et al. [7], but differ from other reported cases due to the degree of bilateral involvement in a relatively elderly female patient. The annular lesion was visible bilaterally and was not occult. In addition, the location of the annular ring was centered around the optic disc, similar to the cases reported by Rodriguez-Coleman et al. and Harino et al. but opposed to other previous cases that were not disc-centered [6, 7]. In our patient, however, the progression was rapid, the visual field deterioration was significant, and the visual field was not well-preserved.

The earlier debate regarding whether AZOOR, and by extension AAOR, is mainly an abnormality of the inner or outer retina has been resolved. Gass et al. have re-evaluated the mechanism of AZOOR after long-term follow-up of AZOOR patients revealed the RPE changes of hypopigmentation and migration [8]. Subsequently, Li et al. described the photoreceptor outer segment dysfunction and degeneration in AZOOR patients [9]. The authors suggested that the fundus changes in AZOOR could be explained by the loss of the outer segment and secondary atrophy of the photoreceptor and RPE [8]. These segments were in corresponding areas of electrophysiological changes and visual field loss. These findings have also been reported by multiple groups [9, 10]. AAOR is part of the spectrum of AZOOR and thus shows similar outer retinal changes. Our case confirms the present the presence of outer retinopathy on multimodal imaging. This case is the first to report the clinical findings in a patient with an associated malignancy of the breast. There were distinct changes at the level of the outer retina and RPE within the demarcated zone. Previously, Tsunoda et al. studied the cone outer segment tip (COST) line in AZOOR and reported selective abnormality in these structures while the photoreceptor inner segment-outer segment junction appeared normal on OCT [11]. The authors found that the COST line appeared indistinct or absent in the region of visual field defect. It is hypothesized that the COST line may be an early indicator of cone photo-receptor dysfunction in patients who present with minimal ophthalmoscopic abnormalities [11]. The demographics and clinical characteristics of published cases of acute annular outer retinopathy is summarized in Table 1.

**Table 1:** Review of literature: demographics and clinical presentation of published cases of acute annular outer retinopathy

**Author, Year**	**No. of cases**	**Age at presentation, years**	**Sex**	**Race**	**Affected Eye**	**Scotoma**	**Duration of follow-up**	**Photopsia**	**Antecedent illness**	**BCVA at presentation**	**RAPD**	**SD-OCT**	**Treatment**	**Fundus findings**	**Visual improvement**	**AC/Vitreous cells**
Dang et al.	1	66	F	White	Both	Present	3 months	Present	N	OS: CF 1ft OD: 20/500	Absent	Normal retinal architecture outside the zone of peripapillary atrophy	Oral prednisone 100 mg daily, taper over 1 month	Distinct and slightly irregular white border along the outer limit of the retinal atrophy	Y	Absent
Seetharam et al. (2015)	1	31	F	White	Right	Present	18 months	Present	N	20/40	Absent	Hyperreflectivity in the ONL and the HFL; marked atrophy of the outer retina within the white ring	None	Distinct white intraretinal line that crossed the macula that extended to the periphery	Y	Absent
Yokoyama et al. (2009)	1	33	F	N/A	Left	Present	15 months	Present	N	20/25	N/A	N/A	Oral prednisone 30 mg daily	OS: Numerous grayish white rings of different sizes peripheral to the vascular arcades; OD: Peripapillary annular grayish white ring	N	N/A
Simunovic et al. (2009)	1	45	F	N/A	Right	Present	1 month	Present	N	20/25	N/A	N/A	N/A	Grey-white peripapillary deep retinal demarcation line	N/A	N/A
Tang et al. (2008)	2	43	F	Asian	Right	Present	2 years	N/A	N	20/50	Present	N/A	Oral prednisolone 60 mg daily, taper over 6 months	Whitish ring around optic disc; larger ring centered over the macula	N	Absent
		35	M	Mixed	Left	Present	2 years	N/A	N	20/20	Present	N/A	Oral prednisone 40 mg daily. Then plasmapheresis + oral cyclophosphamide 150 mg daily, taper over 9 months	OS: Intraretinal whitish-gray ring that traversed 360° of the midperiphery; OD: Whitish intraretinal patch along the temporal retina	Y	N/A
Mitamura et al. (2005) [4]	1	33	F	N/A	Left	Present	1 year	Absent	N	20/30	Present	N/A	None	Irregular, incomplete annular band of grey-white, deep retinal opacification surrounding the optic disc and extending through the foveal centre	Y	Present
Harino et al. (2004) [6]	1	65	M	N/A	Left	Present	2 years	Present	Pneumoconiosis 20 years prior to presentation	20/33	Present	N/A	Kallidinogenase tablets were initiated to improve circulation in the retina and choroid	Lesion surround optic disc well demarcated by a serpentine ring of orange pigment	N	Absent
Cheung et al. (2002) [5]	1	58	F	White	Left	Present	6 weeks	Absent	N	20/30	Present	N/A	Oral prednisolone 60 mg daily + Acyclovir 400 mg 4 times daily	Peripapillary, annular grey-white ring	N	Absent
Fekrat et al. (2000) [3]	4	79	M	White	Left	Present	5 years	Absent	N	20/40	Present	N/A	None	Irregular, imcomplete, annular, white outer retinal ring	Y	Absent
		71	M	White	Right	Present	2 years	Present	N	20/100	Present	N/A	Oral prednisone 60 mg daily, taper over 4 months	Irregular white outer retinal rim	Y	Absent
		29	F	White	Left	Present	6 years	Absent	N	20/25	Absent	N/A	Oral prednisone 60 mg daily, taper over 2 weeks	Irregular, incomplete annular band of white deep retinal opacification	N	Absent
		32	F	White	Left	N/A	1 year	N/A	N	20/200	N/A	N/A	None	Annular lesion around the disk; white outer retinal line along the superonasal edge	Y	N/A
Gass et al. (1995) [1]	1	23	M	N/A	Left	Present	12 years	Absent	N	20/15	Present	N/A	None	Grey retinal ring in the supertemporal quadrant	N	Absent

*AC* anterior chamber, *BCVA* best corrected visual acuity, *RAPD* relative afferent pupillary defect, *SD-OCT* spectral domain optical coherence tomography, *ONL* outer nuclear layer, *HLF* Henle fiber layer

We describe the first patient with the clinical findings of AAOR coinciding with a recent diagnosis of invasive ductal breast carcinoma, raising the question of whether a paraneoplastic syndrome occurred, manifesting as AAOR, based on the close temporal association of symptoms with the diagnosis of breast carcinoma. Our case provides new insight into the early presentation of this uncommon disease and a possible link with a cancer-associated or paraneoplastic etiology of AAOR. Our case differs from most previously reported cases in that our case was bilateral. Further studies are warranted to help elucidate the etiology of the outer retinal changes in patients with AAOR. Ancillary studies, in particular anti-retinal antibody testing and electroretinogram and multifocal electroretinogram, would be helpful in establishing the diagnosis of cancer-associated retinopathy (CAR) if clinical findings demonstrated a paraneoplastic retinopathy. Imaging studies including swept source OCT, OCT angiography, and microperimetry may show characteristic features, progression, and document the course of the disease and its spectrum.

## Data Availability

Data sharing is not applicable to this article as no datasets were generated or analysed during the current study.

## Abbreviations

AAOR: Acute annular outer retinopathy
AZOOR: Acute zonal occult outer retinopathy
BCVA: Best-corrected visual acuity
COST: Cone outer segment tip
RPE: Retinal pigment epithelium
SD-OCT: Spectral-domain optical coherence tomography
